# Management of early recurrence following successful endoscopic detorsion in sigmoid volvulus

**DOI:** 10.12669/pjms.40.9.10078

**Published:** 2024-10

**Authors:** Nurhak Aksungur, Esra Disci, Rifat Peksoz, Sabri Selcuk Atamanalp

**Affiliations:** 1 Nurhak Aksungur, MD Assistant Professor, Department of General Surgery, Faculty of Medicine, Ataturk University, Erzurum, Turkiye; 2 Esra Disci, MD Associate Professor, Department of General Surgery, Faculty of Medicine, Ataturk University, Erzurum, Turkiye; 3 Rifat Peksoz, MD Associate Professor, Department of General Surgery, Faculty of Medicine, Ataturk University, Erzurum, Turkiye; 4 Sabri Selcuk Atamanalp, MD Professor, Department of General Surgery, Faculty of Medicine, Ataturk University, Erzurum, Turkiye

**Keywords:** Sigmoid volvulus, Endoscopic detorsion, Early recurrence

## Abstract

**Objectives:**

Following endoscopic detorsion, sigmoid volvulus (SV) recurs in 3%-86% of patients, approximately 13% of which are early recurrence presenting during the first admission period. Although semielective surgery is the traditional approach, elective surgery following repetitive endoscopy or percutaneous endoscopic sigmoidopexy (PES) are other alternatives in the management of early SV recurrence. Our aim was to discuss the role of semielective surgery in above-mentioned rare clinical entity.

**Methods:**

Among our 1,076-case series, we retrospectively evaluated the records of 612 patients (56.9%) treated between June 1966 and June 1986, while we prospectively utilized the data of 464 patients (43.1%) managed between June 1986 and January 2024. We recorded the treatment option and prognosis for each patient.

**Results:**

Early SV recurrence was determined in 34 (5.5%) of the 621 patients with successful nonoperative detorsion. We treated all of these patients by semielective surgery. The surgical procedures were detorsion in one patient (2.9%), mesopexy in 11 (32.4%), sigmoidectomy with primary anastomosis in 17 (50.0%), and sigmoidectomy with stoma in five (14.7%). In this series, mortality and morbidity rates were 2.9% (one patient) and 14.7% (five patients), respectively.

**Conclusion:**

Semielective surgery is the traditional approach tried by most surgeons, which allows for the recovery of the general status of the patients, bowel preparation, and antibiotic prophylaxis. However, repetitive endoscopy followed by elective surgery or PES are current alternatives for some selected patients. Unfortunately, the relatively low effectuation rate of elective surgery following successful repetitive endoscopic detorsion and recurrence-related poor prognosis are still important handicaps of the latter procedure.

## INTRODUCTION

In sigmoid volvulus (SV), following successful endoscopic detorsion, recurrence rates are relatively high.^1^ In some series, recurrence occurs in seven eighth of SV cases.^2^,^3^ Approximately one eighth of which are early recurrence, that occur during the initial hospitalization period, in the first few hours or days.^4^,^5^ Although some practitioners use surgery and some others prefer repetitive endoscopic detorsion in the treatment of early SV recurrence, the current treatment approach is still controversial.^6^,^7^ To discuss the role of surgery in the management of early SV recurrence, we want to evaluate our 34-patient early recurrence series in a total of 1,076 SV patients treated over 57.5 years. According to the Web of Science^8^ and PubMed^9^ databases, this series constitutes the highest single-center SV data over the world.

## METHODS

A retrospective search was performed to evaluate the medical records of 612 patients (56.9%) treated during a 20 years period (from June 1966 to June 1986), while the data of 464 patients (43.1%) treated in the last 37.5 years (between June 1986 and January 2024) were collected prospectively. The surgical treatment option and prognosis for each patient were recorded.

In our clinical practice, following resuscitation and diagnosis, non-operative detorsion (almost wholly endoscopy) was tried in uncomplicated patients and elective surgery was performed in selected patients with successful nonoperative detorsion. Complicated patients with peritonitis, bowel gangrene or unsuccessful non operative detorsion were treated with emergency surgery. Patients with early SV recurrence were also treated with surgery during the same hospitalization period, which is actually a version of semielective (early elective) surgical management. In the final evaluation, we compared the results of semi elective surgery for early SV recurrence with those of emergency surgery and elective surgery.

### Statistical Analysis:

We used the SPSS v22.0 program (IBM Corporation, Armonk, New York, United States). The data were expressed as mean, standard deviation, minimum, maximum, numbers, or percentages. Numerical variables for more than two groups were compared by using ANOVA test or Kruskal-Wallis test, while categorical variables were compared by using Fisher Freeman Halton test and chi-square test. The significance level was set at p<0.05. All patients or their parents gave informed consents.

### Ethical Approval:

Institutional review board (Ethical Committee of Ataturk University Faculty of Medicine) approved ethical consideration (21.02.2024/68).

## RESULTS

In our 1,076-patient series, nonoperative detorsion was tried in 795 patients (73.9%) including barium enema, rigid endoscopy, and flexible endoscopy in 13, 351, and 431 patients, respectively. In this group, mortality and morbidity rates were 0.6% (five patients) and 2.1% (17 patients) respectively. Endoscopic detorsion was successful in 621 (83.2%) of 746 patients (93.8%) with viable bowels. Early recurrence was determined in 34 patients (5.5%) after a mean 18.8-hour period (range: 6-36 hours). In this group, the mean age was 59.4 years (range: 38-92 years) and the mean American Society of Anaesthesiologists (ASA) score was 2.8 (range: 1-4). The bowels were viable in all the patients, all of whom were treated with semielective surgery. The surgical procedures were detorsion in one patient (2.9%) with an ASA score of 4, mesopexy in 11 (32.4%) with ASA scores of 3-4, and sigmoidectomy in 22 (64.7%) with ASA scores ranging from 1-3. In this group, the mortality and morbidity rates were 2.9% (one patient) and 14.7% (five patients), respectively.

In the presented 1,076-patient series, 488 patients (45.4%) with ASA scores ranging from 1-4 were treated with emergency surgery. The surgical procedures were sigmoidectomy and stoma in 206 patients (42.2%), sigmoidectomy and primary anastomosis in 173 (35.5%), mesopexy in 57 (11.7%), detorsion in 47 (9.6%), sigmoid exteriorization in four (0.8%), and laparotomy in one (0.2%). In this group, the mortality and morbidity rates were 17.4% (85 patients) and 34.2% (167 patients), respectively. When 34 patients with recurrent SV were excluded, among the remaining 454 patients with mean age of 59.2 years (range: 10 weeks-98 years) and ASA scores ranging from 1-4 (mean: 3.2), the mortality and morbidity rates were observed as 18.5% (84 patients) and 35.7% (162 patients), respectively.

In our 1,076-patient series, elective surgery (sigmoidectomy with primary anastomosis by using open procedures in 95 patients and laparoscopic procedures in 21) was performed in 116 (18.7%) of the 621 patients with successful endoscopic detorsion, who had mean age of 56.5 years (range: 18-78) and 1-3 (mean: 2.0) ASA scores. In this group, no mortality was seen, while the morbidity rate was 11.2% (13 patients). As seen in Table-I, the detailed evaluation of the preoperative data demonstrated statistically similar mean ages in all groups (p>0.05).

**Table-I:** Statistical analyses in patients with sigmoid volvulus.

Parameter	Semi-elective surgery	Emergency surgery	Elective surgery	Statistical analysis
Total	34	454	116	-
Age	59.4 (38-92)	59.2 (10 w-98)	56.5 (18-78)	ANOVA test F=0.396; p=0.755
ASA	2.8^a^ (1-4)	3.2^a^ (1-4)	2.0^b^ (1-3)	ANOVA test F=35.444; p<0.001
Mortality	1^a,b^ (2.9%)	84^a^ (18.5%)	0^b^ (0.0%)	Fisher Freeman Halton test x^2^=45.062; p<0.001
Morbidity	5^a,b^ (14.7%)	162^a^ (35.7%)	13^b^ (11.2%)	Chi-square test x^2^=36.111; p<0.001

Similarly, the preoperative ASA scores were statistically similar between semielective surgery and emergency surgery groups (p>0.05), while the mean ASA score was significantly lower in the elective surgery group (p<0.001). Regarding the evaluation of the postoperative data, the mortality rate of the semielective surgery group were statistically similar when compared with that of emergency surgery and elective surgery groups (p>0.05).

However, the mortality rate of the elective surgery group was significantly lower when compared with that of the emergency surgery group (p<0.001). Similarly, the morbidity rate of the semielective surgery group were statistically similar when compared with that of emergency surgery and elective surgery groups (p>0.05). However, the morbidity rate of the elective surgery group was significantly lower when compared with that of the emergency surgery group (p<0.001).

## DISCUSSION

In SV, first-line treatment is endoscopic detorsion in patients in whom peritonitis, bowel gangrene or perforation is not present.^1^,^7^ Following successful endoscopic detorsion, SV recurs in 3%-86% of patients, approximately 13% of which occur during the initial hospitalization period, in the first few hours or days.^1^-^5^ However, there is no laboratory, radiological or clinical parameter accurately indicating the occurrence of SV recurrence in any patient.^10^

Most likely, due to the relatively low incidence of early SV recurrence, the treatment algorithm is not clearly identified.^6^,^7^ Most practitioners prefer surgery, which is actually a version of semielective (early elective) surgical management, that allows for the improvement of the performance status of patients, bowel preparation, and antibiotic prophylaxis. On the other hand, some other clinicians prefer repetitive endoscopic trials, which provides elective surgery in selected non-elderly and well-conditioned patients, while percutaneous endoscopic sigmoidopexy (PES) may be an alternative procedure in some elderly and bad-conditioned individuals.^11^,^12^ An electronic data search of the last 79 years of literature (from 1945 to 2024) revealed only a few reports on this issue.^8^,^9^ Bruzzi et al.^13^ reported a relatively large series on early SV recurrence including 22 patients. They used endoscopic procedures in 11 patients including endoscopic sigmoidopexy in six cases and endoscopic detorsion in five, while the remaining 11 patients were treated with surgery including sigmoidectomy in seven cases, sigmoidopexy in three, and sigmoid colostomy in one.

However, Johansson et al.^14^ and Mulas et al.^15^ preferred emergency surgery in their four- and three-case series, respectively. Similarly, Kim et al.^16^ reported a patient treated with emergency surgery, while Colinet et al.^17^ performed sigmoidectomy in six cases. Conversely, Iida et al.^18^ tried repetitive endoscopy in six patients, five of whom were treated with elective sigmoidectomy following successful endoscopy. Similarly, Maddah et al.^19^ applied a second endoscopic procedure in four patients and Lou et al.^20^ applied the same procedure in their six-patient series. On the other hand, Garrido et al.^21^ performed PES following repetitive endoscopic detorsion. However, there are no clear results on the success, complication, or recurrence rates of both surgery and repetitive endoscopic detorsion for early SV recurrence in above-mentioned reports.

Bowel gangrene clearly develops in 6.1-30.2% of SV patients, which is a heavy prognostic factor.^22^ However, our study demonstrated that early diagnosis via medical observation may improve bowel viability in patients with early SV recurrence. In semielective surgery following SV recurrence, a preoperative preparation phase is obtained between nonoperative detorsion and surgery following recurrence. This practice allows for bowel preparation or provides an empty colon in addition to antibiotic prophylaxis. This preoperative preparation reduces postoperative mortality and morbidity in patients with sigmoidectomy.^23^

Moreover, due to the old age preference of SV, approximately one-fourth of SV patients have comorbidities.^24^ The above mentioned preoperative period may also provide an opportunity for the assessment and recovery of the general status of patients. In the end, early SV recurrence under medical observation may provide some advantages when compared with that of primary SV, as was demonstrated in our study. Regarding the surgical technique, sigmoidectomy with primary anastomosis or a recurrence-reducing procedure such as sigmoidopexy, mesopexy, mesoplasty, or extraperitonealization are the preferred options.^3^,^12^,^25^

However, the relatively high mortality (2.9% vs. 0.0%) and morbidity (14.7% vs. 11.2%) rates in our series compared with those of elective surgery may restrict the usage of semielective surgery in some cases. As an alternative, elective surgery following repetitive endoscopic detorsion during the same hospitalization period may be used in selected patients.^12^,^25^ Our study demonstrated that the best results are obtained by elective surgery, preferably by laparoscopic procedures following successful endoscopic detorsion, in which the mortality rate is less than 2% and the morbidity rate is approximately 5-15%.^2^,^5^,^8^,^12^,^25^

However, most patients fail to return for elective surgery following discharge.^1^,^9^ Similar to the literature findings, the relatively low effectuation rate of elective surgery in our series (18.7%) has drawn the attention of practitioners.^2^,^11^,^12^ In clinical practice, approximately one fourth of such patients reapply to a health center with approximately one fifth of them with bowel gangrene.^5^,^9^ Finally, PES may be an option for select patients with severe conditions and elderly patients.^24^,^25^

As a result, based on obtained literature data and our experiments, semielective surgery is the most preferred option in the treatment of early SV recurrence. However, the relatively high mortality and morbidity rates of this procedure when compared with that of elective surgery forces the practitioners’ hand to perform repetitive endoscopic detorsion followed by elective surgery in non-elderly and well-conditioned patients, while PES in elderly and bad-conditioned individuals. Unfortunately, the relatively low effectuation rate of elective surgery following successful repetitive endoscopic detorsion and recurrence-related poor prognosis arising from bowel gangrene are still important handicaps. Despite everything, at least in some selected patients, elective surgery following repetitive endoscopy or PES may be our new targets instead of traditional practice, semielective surgery.

### Limitations:

The relative rare incidence of early SV recurrence in addition to the use of semielective surgery alone as a unique treatment option and the partial retrospective evaluation of the data are the main limitations of the present study. New prospective controlled clinical studies including other treatment options such as repetitive endoscopy followed by elective surgery or PES may give new information about new treatment algorithms. However, large-series studies require tens of patients and decades, which is quite difficult in patients with uncommon clinical conditions such early SV recurrence.

## CONCLUSIONS

Following endoscopic detorsion, sigmoid volvulus (SV) recurs in 3%-86% of patients, and approximately 13% of cases occur during the first admission period. In the treatment of early SV recurrence, some practitioners prefer surgery, while others use repetitive endoscopic detorsion. Most likely due to its relatively low incidence, the current treatment approach for early SV recurrence is still controversial. Although semielective surgery is the traditional approach tried by most surgeons, repetitive endoscopy followed by elective surgery or PES are other alternatives.

### Authors’ Contribution:

**NA and SSA:** Data collection, manuscript writing.

**ED and RP:** Data collection, revision of the final draft.

**SSA:** Is responsible and accountable for the accuracy or integrity of the work.
